# Long non-coding RNA SNHG10 upregulates BIN1 to suppress the tumorigenesis and epithelial–mesenchymal transition of epithelial ovarian cancer via sponging miR-200a-3p

**DOI:** 10.1038/s41420-022-00825-9

**Published:** 2022-02-11

**Authors:** Wei Lv, Yunlong Jia, Jiali Wang, Yuqing Duan, Xuexiao Wang, Tianxu Liu, Shuwei Hao, Lihua Liu

**Affiliations:** 1 Department of Tumor Immunotherapy, Fourth Hospital of Hebei Medical University and Hebei Cancer Institute, 050035 Shijiazhuang, China; 2 Department of Gynecology, Fourth Hospital of Hebei Medical University and Hebei Cancer Institute, 050035 Shijiazhuang, China; 3 International Cooperation Laboratory of Stem Cell Research, Hebei Medical University, 050017 Shijiazhuang, China

**Keywords:** Tumour-suppressor proteins, Ovarian cancer, Long non-coding RNAs

## Abstract

Epithelial ovarian cancer (EOC) is one of the most frequent and fatal gynecologic malignant tumors resulting in an unsatisfying prognosis. Long non-coding RNAs (lncRNAs) play pivotal roles in the tumorigenesis and progression of EOC. However, the profile of lncRNAs involved in EOC remains to be expanded to further improve clinical treatment strategy. In present study, we identified a novel tumor-suppressive lncRNA small nucleolar RNA host gene 10 (SNHG10) in EOC. Kaplan–Meier analysis and COX proportional hazard progression model showed that low expression of SNHG10 was correlated with a poor prognosis of EOC patients. Overexpressing SNHG10 suppressed the proliferation, colony formation, migration, and invasion of EOC cells. Furthermore, SNHG10 was predicted to sponge miR-200a-3p in EOC cells according to the LncBase v.2 experimental module. Then, the binding of SNHG10 and miR-200a-3p was confirmed by performing quantitative real-time PCR (qRT-PCR) and luciferase reporter assays. RNA immunoprecipitation (RIP) showed that SNHG10 and miR-200a-3p occupied the same Ago2 protein to form an RNA-induced silencing complex (RISC). By overlapping the results from the bioinformatics algorithms, tumor-suppressor bridging integrator-1 (BIN1) was found to be a main downstream target of the SNHG10/miR-200a-3p axis. Low expression of BIN1 in EOC tissues was detected by using immunohistochemistry (IHC). Besides, BIN1 and SNHG10 expression was positively correlated in EOC tissues. By performing miRNA rescue experiments, a SNHG10/miR-200a-3p/BIN1 axis and its promoting effects on malignant behaviors and epithelial–mesenchymal transition (EMT) process were verified in EOC cells. Moreover, SNHG10 overexpression significantly suppressed the tumorigenesis and EMT of EOC cells in vivo. Altogether, SNHG10 sponges miR-200a-3p to upregulate BIN1 and thereby exerting its tumor-suppressive effects in EOC. Therefore, the SNHG10/miR-200a-3p/BIN1 axis may act as a potential predictive biomarker and therapeutic target for treating EOC.

## Introduction

Ovarian cancer is one of the most gynecological malignant tumors worldwide, and is characterized by an uncertain clinical manifestation and poor outcome [1]. Epithelial ovarian cancer (EOC) is the most common and fatal subtype of ovarian cancer [2]. The treatment strategy of EOC is mainly based on three pillars: cytoreductive surgery, platinum-based chemotherapy, and targeted therapies. Despite advances in the treatment concepts and methods, the overall prognosis of EOC patients has not been well improved [3]. The molecules involved in the tumorigenesis and progression of EOC remain elusive, limiting advances in clinical treatment. Therefore, further clarifying the molecules correlated with EOC is of great significance improving patients’ prognosis.

Long non-coding RNAs (lncRNAs) are a class of non-coding transcripts longer than 200 nucleotides (nt) [4]. Accumulating studies show that lncRNAs participate in multiple cellular processes by interacting with macromolecules, including DNA, RNA, chromatin, and proteins [4]. Emerging evidence indicates that lncRNAs are more tissue-specific than protein-coding genes, and different cancers have some specific lncRNA expression signatures [5]. Numerous lncRNAs have been proven to be involved in regulating the malignant biological behaviors of cancer cells, such as proliferation, invasion, metastasis, immune escape and treatment resistance [6]. For instance, small nucleolar RNA host gene 18 (SNHG18) promotes the proliferation and metastasis of non-small cell lung cancer (NSCLC) by regulating the expression of bromodomain containing 4 (BRD4) [7]. Linc00312 impairs DNA damage repair to suppress the radiotherapy resistance in nasopharyngeal carcinoma cells by hindering the recruitment of DNA-PKcs to Ku80 [8]. LncRNA UCA1 promotes the immune escape of gastric cancer by upregulating programmed death ligand 1 (PD-L1) [9]. LncRNAs also play crucial roles in the tumorigenesis and progression of EOC, and some lncRNAs have the potential to be novel biomarkers for predicting the prognosis of EOC patients [10]. However, the profile of lncRNA involved in EOC still needs to be complemented.

SNHG10 is a novel lncRNA whose function in EOC remain unknown. Herein, we identified SNHG10 as a downregulated lncRNA in EOC tissues. Overexpressing SNHG10 suppressed the proliferation, colony formation, migration, and invasion of EOC cells. In addition, we found that SNHG10 sponged miR-200a-3p to regulate the expression of bridging integrator-1 (BIN1), a tumor-suppressor downregulated in numerous cancers. Moreover, the suppressive effect of the SNHG10/miR-200a-3p/BIN1 axis on epithelial–mesenchymal transition (EMT) in EOC cells was verified. Subsequently, the inhibitory effect of SNHG10 on tumorigenesis and EMT in vivo was detected in a xenograft mouse model. Furthermore, SNHG10 and BIN1 were found to be positively correlated with the prognosis of EOC patients. In summary, the SNHG10/miR-200a-3p/BIN1 axis might be a novel predictive biomarker and therapeutic target for treating EOC.

## Results

### SNHG10 expression is downregulated in EOC and correlates with poor prognosis

**Fig. 1:**
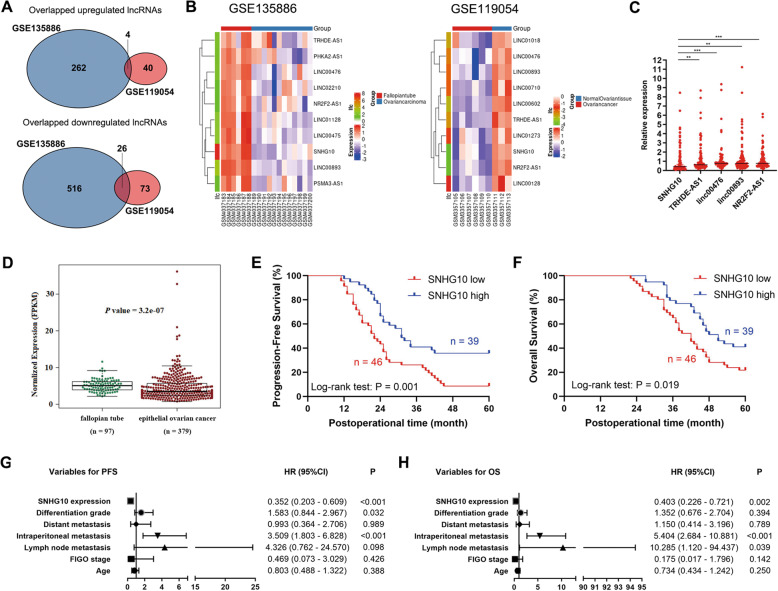
SNHG10 is downregulated in EOC tissues and positively correlated with prognosis of patients. **A** Schematic illustrations showing the overlapped lncRNAs in GSE135886 and GSE119054 dataset. **B** The top 10 downregulated lncRNAs in GEO dataset GSE135886 and GSE119054. **C** The expressions of the overlapped downregulated lncRNAs in EOC specimens. **D** SNHG10 exhibited lower expression in EOC tissues (*N* = 97), compared to the fallopian tube (*N* = 379), by analyzing the TCGA database. **E** SNHG10 expression was positively correlated with the PFS of EOC patients, analyzed by Kapan–Meier analysis. **F** SNHG10 expression was positively correlated with the OS of EOC patients, analyzed by Kapan–Meier analysis. **G** Variables predicting PFS of EOC patients, analyzed by COX proportional hazard progression model. **H** Variables predicting the OS of EOC patients, analyzed by COX proportional hazard progression model. *P < 0.05, **P < 0.01, ***P < 0.001.

To verify the lncRNAs involved in EOC tumorigenesis and progression, we analyzed the EOC-related lncRNA profile in the Gene Expression Omnibus (GEO) database by R. First, we analyzed the GSE135886 and GSE119054 [11, 12], we found well-annotated lncRNAs in EOC (Supplementary Fig. 1A, B). Then, we overlapped these results and found that 26 lncRNAs were downregulated and 4 lncRNAs were upregulated in EOC tissues (Fig. 1A and Supplementary Table S3). Aiming at verifying the critical tumor-suppressing lncRNAs, we further analyzed the top 10 downregulated lncRNAs in EOC tissues of GSE135886 and GSE119054 datasets with R. The overlapped results showed that there were 5 common lncRNAs (SNHG10, TRHDE-AS1, linc00476, linc00893, NR2F2-AS1) (Fig. 1B). Then we detected the expression of these 5 lncRNAs by quantitative real-time PCR (qRT-PCR). The results showed that all these five lncRNAs were expressed at low levels, and SNHG10 showed the lowest expression (Fig. 1C). Then, we explored SNHG10 expression from The Cancer Genome Atlas (TCGA) database and found that SNHG10 expression was significantly downregulated in EOC (*P* < 0.001, Fig. 1D). Therefore, we then analyzed the clinical significance of SNHG10 in further study. The cut-off value of SNHG10 for analyzing progression-free survival (PFS) and overall survival (OS) was 0.550, calculated according to the expression level of SNHG10, PFS/OS time, and progression/survival status by using X-tile [13]. Kaplan–Meier analysis displayed that low expression of SNHG10 was correlated with shorter PFS and OS (*P* = 0.001, *P* = 0.019; Fig. 1E, F). Then, the COX proportional hazard regression model was applied to assess the role of SNHG10 in predicting prognosis of EOC patients. The results showed that intraperitoneal metastasis and SNHG10 expression were identified to be significantly correlated with PFS in EOC patients (Fig. 1G). In addition, lymph node metastasis, intraperitoneal metastasis and SNHG10 expression were found to be significantly correlated with the OS of EOC patients (Fig. 1H). The above results indicated that low expression of SNHG10 was significantly correlated with the prognosis of EOC, and that SNHG10 expression might be regarded as a potential independent predictive biomarker for EOC patients.

### SNHG10 suppresses the proliferation, migration, and invasion of EOC cells in vitro

**Fig. 2:**
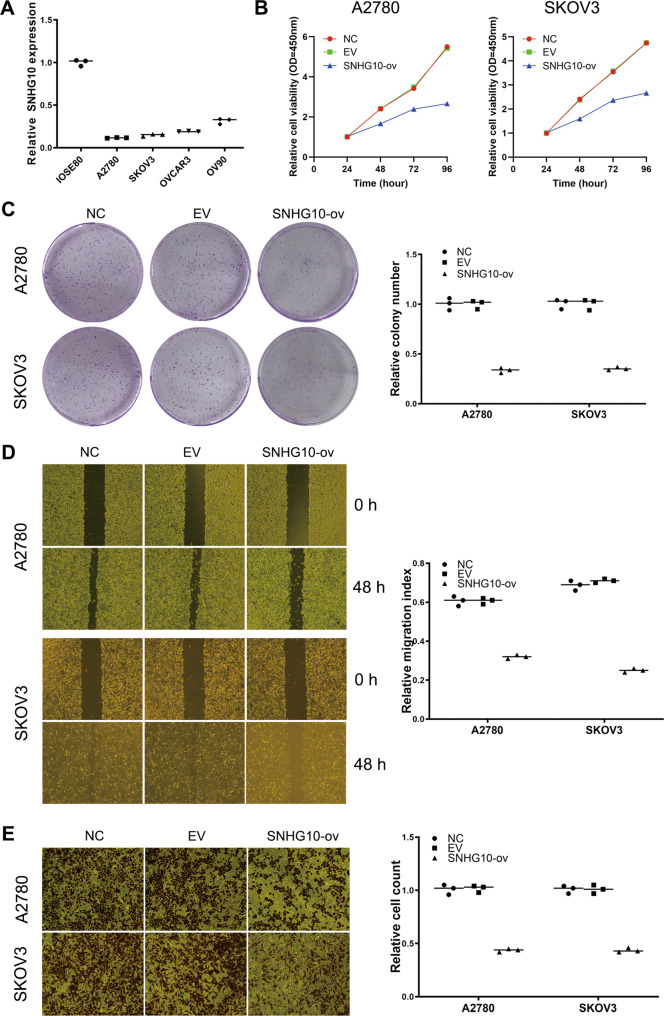
SNHG10 overexpression suppresses the proliferation, migration, and invasion ability of EOC cells in vitro. **A** Expression of SNHG10 in EOC cell lines, assayed by qRT-PCR, normal ovarian epithelial cell line IOSE80. **B** SNHG10 overexpression suppressed proliferation ability of EOC cells, assayed by CCK-8 method. Plots indicate mean. **C** SNHG10 overexpression suppressed colony formation ability in EOC cells. **D** SNHG10 overexpression suppressed migratory ability in EOC cells, assayed by wound-healing experiment. **E** SNHG10 overexpression suppressed invasive ability in EOC cells, assayed by transwell. The experiments were conducted in triplicate.

Given that SNHG10 was significantly correlated with a poor prognosis in EOC patients, we next evaluated its biological functions. We first detected the expression of SNHG10 in EOC cell lines. The results showed that SNHG10 expression was significantly lower in EOC cell lines than in the IOSE80 cells (Fig. 2A). Additionally, A2780 and SKOV3 cells showed the lowest expression of SNHG10 among these EOC cell lines. To explore the functional roles of SNHG10, we then established A2780 and SKOV3 cells that stably overexpressed SNHG10. By performing CCK-8 assay, colony formation assay, wound-healing experiments and transwell Matrigel assay we found that the proliferation, colony formation, migration and invasion of A2780 and SKOV3 cells were significantly decreased by SNHG10 overexpression, compared to the empty vector (EV) group (Fig. 2B–E). Then, to further identify the biological role of SNHG10 in EOC, we used siRNAs to knock down SNHG10 in A2780 and SKOV3 cells. As shown in Supplementary Fig. 2A–E, si-SNHG10#1 and si-SNHG10#2 significantly reduced the expression of SNHG10 and slightly enhanced the proliferation, invasion, and migration of A2780 and SKOV3 cell. Taken together, SNHG10 suppressed the proliferation, colony formation, migration, and invasion of EOC cells.

### SNHG10 acts as a competing endogenous RNA (ceRNA) for sponging miR-200a-3p in EOC

**Fig. 3:**
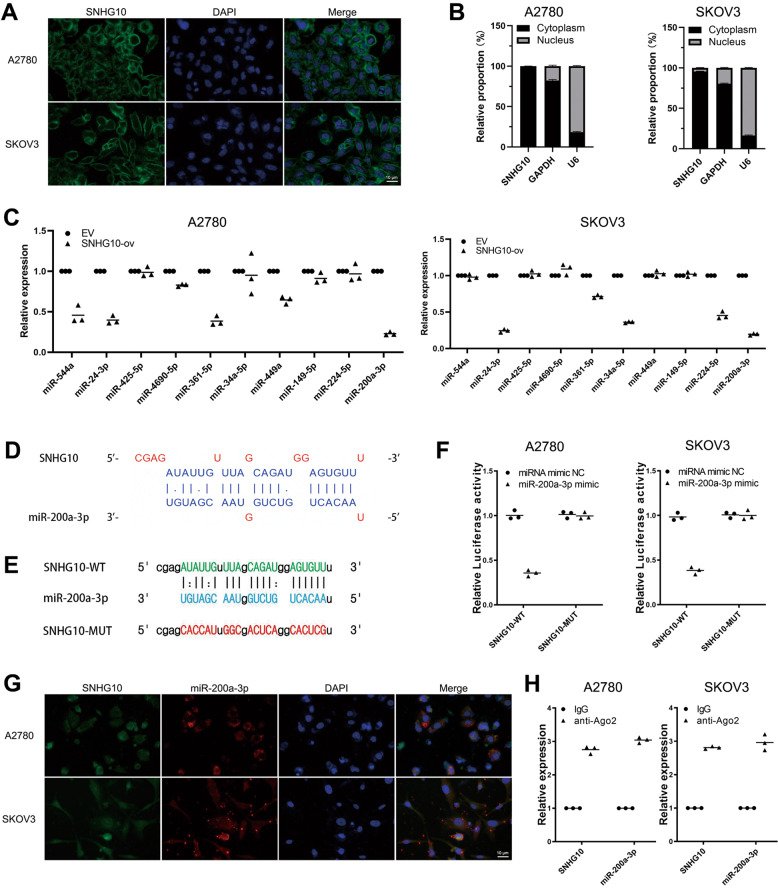
SNHG10 sponges miR-200a-3p in EOC cells. **A** SNHG10 located in the cytoplasm of EOC cells. **B** The nuclear and cytoplasmic distribution of SNHG10 in A2780 and SKOV3 cells. **C** Altered expressions of miRNAs in A2780 cells after overexpressing SNHG10. **D** The predicted binding between miR-200a-3p and SNHG10, analyzed with LncBase v.2 experiment module. **E** The sequences of SNHG10-WT and SNHG10-MUT. **F** miR-200-3p mimic decreased the luciferase activity in EOC cells transfected with SNHG10-WT but not in the cells transfected with SNHG10-MUT. **G** SNHG10 and miR-200a-3p were co-located in the cytoplasm of EOC cells. **H** RIP was performed using AGO2 antibody in A2780 and SKOV3 cells, the enrichments of SNHG10 and miR-200a-3p were detected by qRT-PCR. The experiments were conducted in triplicate.

Emerging studies have shown that cytoplasmic lncRNAs can act as sponges for miRNAs to regulate their downstream targets in cancers [6]. To explore whether SNHG10 functioned as a ceRNA to sponge miRNAs, we detected the subcellular location of SNHG10 in EOC cells by FISH. As shown in Fig. 3A, SNHG10 was mainly located in the cytoplasm. Furthermore, we conducted nuclear and cytoplasmic fraction isolation experiments and qRT-PCR to determine the subcellular distribution of SNHG10 in EOC. As shown in Fig. 3B, the cytoplasmic proportion of SNHG10 was 97.880 ± 0.155% and 95.443 ± 0.482% in A2780 and SKOV3 cells, respectively. Therefore, the abundance of nuclear SNHG10 was not sufficient to play biological functions. Then, we used the LncBase v.2 experimental module to predict the potential miRNAs binding with SNHG10 [14, 15]. The top ten possible miRNAs are listed in Supplementary Table S4. Then, we transfected SNHG10 into A2780 and SKOV3 cells and detected the expression of these candidate miRNAs. Among the ten candidate miRNAs, miR-544a, miR-24-3p, miR-361-5p, miR-449a and miR-200a-3p were downregulated in A2780 cells, miR-24-3p, miR-361-5p, miR-34a-5p, miR-224-5p and miR-200a-3p were downregulated in SKOV3 cells (*P* < 0.05, Fig. 3C). Taken together, miR-24-3p, miR-361-5p and miR-200a-3p were the overlapped miRNAs. As Fig. 3D and Supplementary Fig. 3A demonstrates, there were potential binding sites on the SNHG10 transcript for interaction with miR-200a-3p, miR-24-3p, and miR-361-5p. To verify whether SNHG10 could bind with these miRNAs, luciferase reporters containing wild-type (SNHG10-WT) and mutated-binding sites (SNHG10-MUT) for miRNAs were constructed and transfected into A2780 and SKOV3 cells (Fig. 3E and Supplementary Fig. 3B). The results demonstrated that cotransfection of three miRNAs mimic significantly reduced the luciferase activity in A2780 and SKOV3 cells transfected with SNHG10-WT, but not in those transfected with SNHG10-MUT (Fig. 3F and Supplementary Fig. 3C). As the reduction in miR-200a-3p expression was the most significant (*P* < 0.001, Fig. 3B), we selected miR-200a-3p in subsequent studies.

To further evaluate the potential binding of SNHG10 and miR-200a-3p, we detected their subcellular location in A2780 and SKOV3 cells by FISH. The results showed that SNHG10 and miR-200a-3p were colocalized in the cytoplasm (Fig. 3G). Then, we conducted a RNA immunoprecipitation (RIP) assay with an anti-Argonaute-2 (Ago2) antibody. The expressions of SNHG10 and miR-200a-3p were significantly higher in Ago2 pellets than in IgG pellets (Fig. 3H). These findings suggested that SNHG10 acted as a ceRNA for miR-200a-3p and formed an RNA-induced silencing complex (RISC) in EOC cells.

**Fig. 4:**
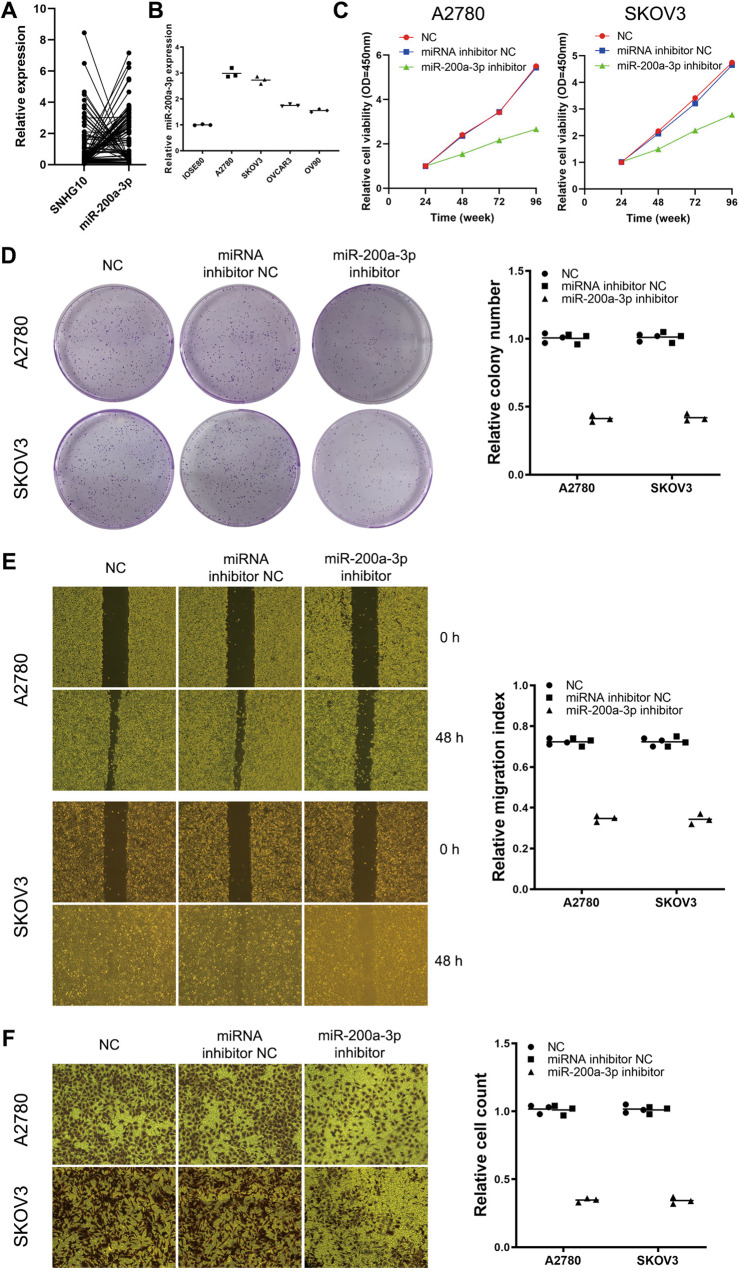
miR-200a-3p inhibitor suppresses the proliferation, migration, and invasion of EOC cells in vitro. **A** Correlation between SNHG10 and miR-200a-3p in EOC tissues (*N* = 85). **B** The expression of miR-200a-3p in EOC cell lines, normal ovarian epithelial cell IOSE80 as control. **C** miR-200a-3p inhibitor suppressed the proliferation ability in EOC cells, assayed by CCK-8 method. Plots indicate mean. **D** miR-200a-3p inhibitor suppressed the colony formation ability in EOC cells. **E** miR-200a-3p inhibitor suppressed the migration ability in EOC cells, assayed by wound-healing experiment. **F** miR-200a-3p inhibitor suppressed the invasion ability in EOC cells, assayed by transwell. The experiments were conducted in triplicate.

Since the biological significance of miR-200a-3p in EOC tumorigenesis and progression is still disputed [16, 17], we then detected the expression of miR-200a-3p in EOC specimens and analyzed its correlation with SNHG10. The results showed that SNHG10 and miR-200a-3p was negatively correlated (*r* = −0.286, *P* = 0.008; Fig. 4A). Next, we studied the effects of miR-200a-3p on the malignant behaviors to determine whether it acted as a tumor-promoting miRNA in EOC. A2780, SKOV3, OVCAR3 and OV90 cells exhibited higher expression of miR-200a-3p than IOSE80 cells (all *P* < 0.001; Fig. 4B). Then, we further used a miR-200a-3p inhibitor to determine its effect on the malignant behaviors in A2780 and SKOV3 cells. By performing CCK-8 assay, colony formation assay, wound-healing experiments and transwell Matrigel assay we found that miR-200a-3p inhibitor suppressed the proliferation, colony formation, migration and invasion of A2780 and SKOV3 cells (Fig. 4C–F). Taken together, these findings indicated that the miR-200a-3p inhibitor suppressed numerous malignant behaviors in A2780 and SKOV3 cells, indicating that miR-200a-3p might be a tumor-promoting gene in EOC. Thus, SNHG10 might serve as a tumor-suppressing lncRNA in EOC by sponging miR-200a-3p.

### BIN1 is a target of the SNHG10/miR-200a-3p axis in EOC cells

**Fig. 5:**
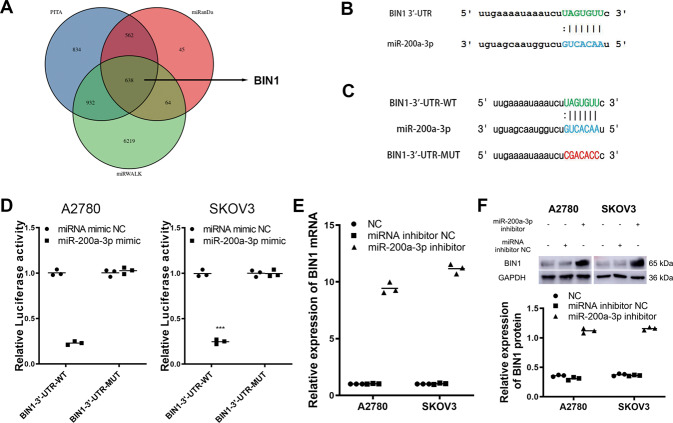
BIN1 is a direct target of miR-200a-3p in EOC cells. **A** BIN1 was contained in the overlapped result from the bioinformatic algorithm PITA, miRanDa and miRWALK. **B** The predicted binding between miR-200a-3p and BIN1-3’-UTR. **C** The sequences of BIN1-3’-UTR-WT and BIN1-3’-UTR-MUT. **D** miR-200-3p mimic decreased the luciferase activity of EOC cells transfected with BIN1-3’-UTR-WT but not in the cells transfected with BIN1-3’-UTR-MUT. **E** The miR-200a-3p inhibitor increased the expression of BIN1 mRNA in EOC cells. **F** The miR-200a-3p inhibitor increased the expression of BIN1 mRNA in EOC cells. The experiments were conducted in triplicate.

To explore the mechanism of the SNHG10/miR-200a-3p axis in EOC, we used the bioinformatics algorithms PITA, miRanDa and miRWALK to predict the potential targets. By overlapping the results, we found 638 possible genes regulated by miR-200a-3p in cancers (Fig. 5A and Supplementary Table S5). Among these genes, BIN1 caught our attention due to its tumor-suppressing effect revealed by our previous studies [18–22]. As shown in Fig. 5B, there was a potential binding site on BIN1 for interacting with miR-200a-3p. Then, we performed luciferase assays to determine the effect of miR-200a-3p on BIN1. We established a luciferase plasmid harboring wild type 3’-UTR sequence of BIN1 (BIN1-3’-UTR-WT) and co-transfected A2780 and SKOV3 cells with the miR-200a-3p mimic. Meanwhile, a reporter vector containing a mutation in the miR-200a-3p-binding sites on the 3’-UTR sequence of BIN1 (BIN1-3’-UTR-MUT) was also established and co-transfected into A2780 and SKOV3 cells (Fig. 5C). Transfection of the miR-200a-3p mimic significantly reduced the luciferase activity in A2780 and SKOV3 cells transfected with BIN1-3’-UTR-WT, but not in those transfected with BIN1-3’-UTR-MUT (Fig. 5D). Furthermore, transfection of miR-200a-3p inhibitor significantly increased the expression of BIN1 mRNA in A2780 and SKOV3 cells (Fig. 5E). Consistent with qRT-PCR results, the miR-200a-3p inhibitor significantly increased the BIN1 protein expression in A2780 and SKOV3 cells, as detected by western blotting (Fig. 5F). These results indicated that miR-200a-3p decreased the expression of BIN1 in A2780 and SKOV3 cells.

**Fig. 6:**
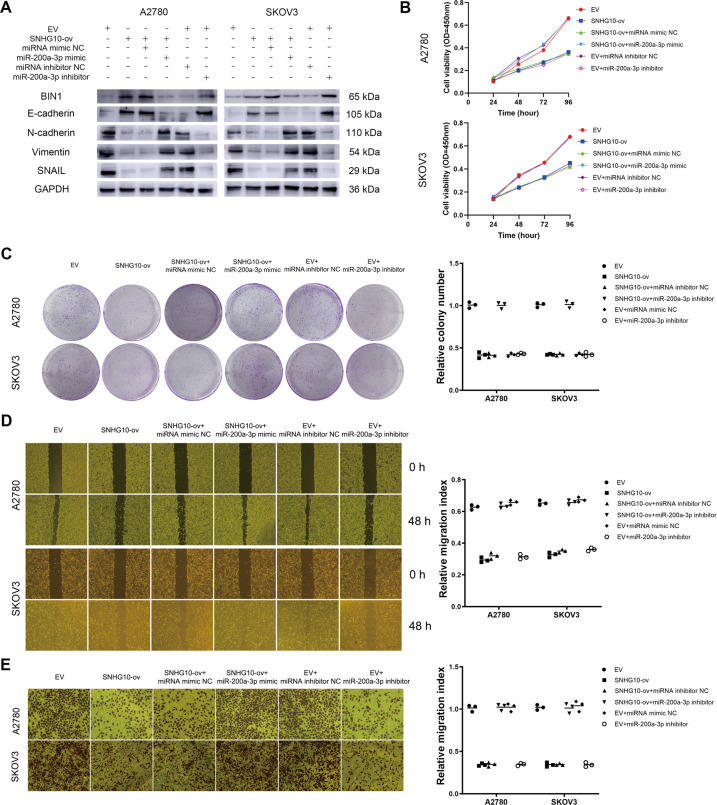
The effects of SNHG10/miR-200a-3p/BIN1 axis on the malignant behaviors of EOC cells. **A** The effects of the SNHG10/miR-200a-3p axis on the expressions of BIN1 and EMT-related proteins. **B** The effects of the SNHG10/miR-200a-3p/BIN1 axis on the proliferation ability in EOC cells. **C** The effects of the SNHG10/miR-200a-3p/BIN1 axis on the clone formation ability in EOC cells. **D** The effects of the SNHG10/miR-200a-3p/BIN1 axis on the migration ability in EOC cells. **E** The effects of the SNHG10/miR-200a-3p/BIN1 axis on the invasion ability in EOC cells. The experiments were conducted in triplicate.

Next, we detected the effect of the SNHG10/miR-200a-3p axis on BIN1 expression. The results showed that overexpression of SNHG10 increased BIN1 expression while miR-200a-3p rescued this upregulation (Fig. 6A). Since our previous study revealed that BIN1 suppressed EMT in esophageal squamous cell carcinoma (ESCC) [20], we then verified whether the SNHG10/miR-200a-3p axis affected EMT-related proteins by regulating BIN1 in A2780 and SKOV3 cells. The results showed that the SNHG10 overexpression led to increased expression of E-cadherin and decreased expression of N-cadherin, Vimentin and SNAIL, while cotransfection of miR-200a-3p mimic neutralized this phenomenon (Fig. 6A). SNHG10 overexpression significantly inhibited the proliferation, colony formation, migration and invasion of A2780 and SKOV3 cells, while these effects were abolished by cotransfection of miR-200a-3p mimic (Fig. 6B–E).

**Fig. 7:**
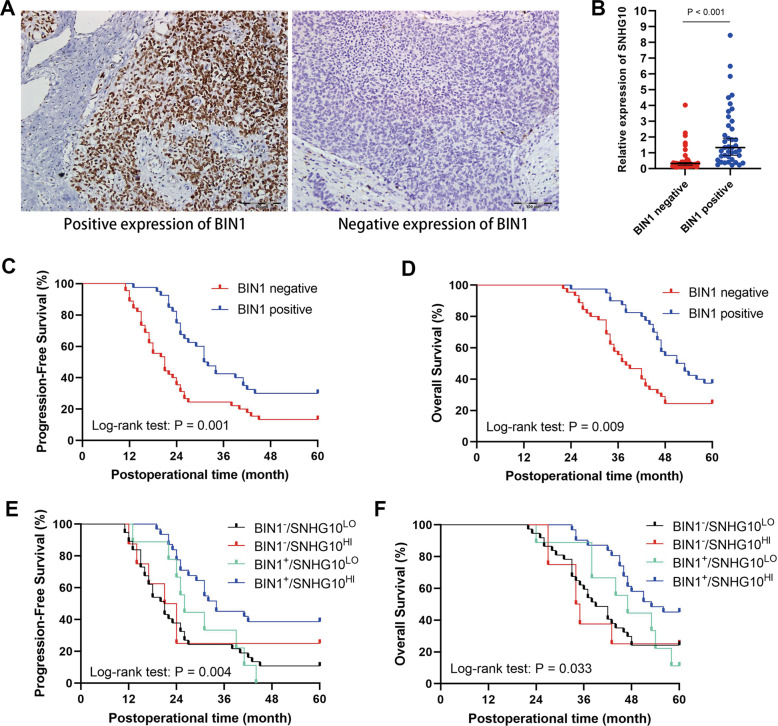
Clinical significance of BIN1 in EOC. **A** Presentative staining of BIN1 protein in EOC tissues, detected by IHC (×400). **B** The expression of SNHG10 was lower in BIN1-negative EOC tissues (*N* = 45), compared to BIN1-positive EOC tissues (*N* = 40). **C** BIN1 expression was positively correlated with the PFS of EOC patients. **D** BIN1 expression was positively correlated with the OS of EOC patients. **E** Co-expression of SNHG10 and BIN1 expression positively correlated with the PFS of EOC patients. **F** Co-expression of SNHG10 and BIN1 expression positively correlated with the OS of EOC patients. The experiments were conducted in triplicate.

We next detected BIN1 expression in EOC specimens by immunohistochemistry (IHC) and analyzed its correlation with prognosis. BIN1 staining was mainly located in the nucleus of cancer cells in EOC tissues (Fig. 7A). Among 85 EOC specimens, 45 (52.94%) showed low expression of BIN1. The SNHG10 expression in the tumor tissues with positive expression of BIN1 was significantly higher, than that in the tissues with negative expression of BIN1, indicating that SNHG10 was positively correlated with BIN1 (*P* < 0.001, Fig. 7B). Then, we analyzed the correlation between BIN1 expression and the prognosis of EOC patients. Kaplan–Meier analysis showed that negative expression of BIN1 was significantly correlated with shorter PFS and OS (log-rank test: *P* = 0.001, *P* = 0.009; Fig. 7C, D). Next, we divided the EOC patients into four groups (i.e., SNHG10-low/BIN1-positive, SNHG10-low/BIN1-negative, SNHG10-high/BIN1-positive and SNHG10-high/BIN1-negative) according to the expression status of SNHG10 and BIN1, and then performed Kaplan–Meier analysis to reveal the clinical significance of the co-expression of SNHG10 and BIN1. The data showed that the PFS and OS of the SNHG10-high/BIN1-positive group were significantly longer than those of the other three groups (log-rank test: *P* = 0.004, *P* = 0.033; Fig. 7E, F). Taken together, these data provided evidence for the tumor-suppressing role of the SNHG10/miR-200a-3p/BIN1 axis in EOC.

### SNHG10 inhibits the tumorigenesis and progression of EOC in vivo

**Fig. 8:**
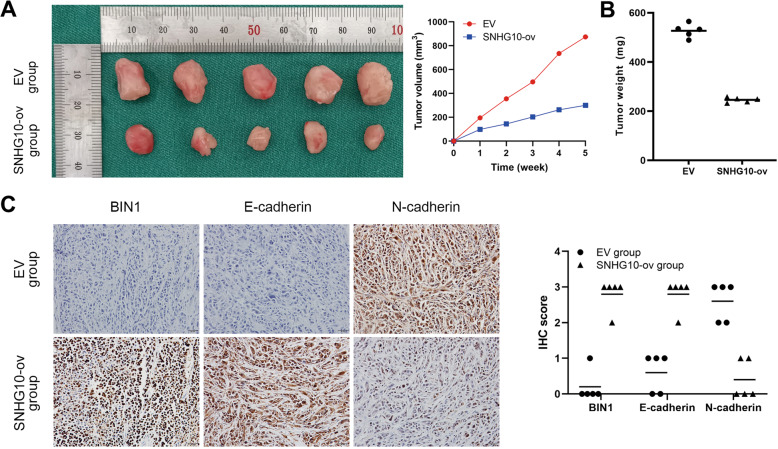
The effects of SNHG10 on EOC cells in vivo. **A** SNHG10 overexpression significantly decreased the tumor volume of tumor-bearing mouse model (*N* = 5). Plots indicate mean. **B** SNHG10 overexpression significantly decreased the tumor weight of tumor-bearing mouse model (*N* = 5). **C** Difference in the expression of BIN1, E-cadherin and N-cadherin in EOC tissue of xenograft mouse model between EV group and SNHG10-ovexpression group, detected by IHC (*N* = 5).

To confirm whether the SNHG10/miR-200a-3p/BIN1 axis exerted tumor-suppressing functions in vivo, we established a xenograft tumor mouse model. Ten female BALB/c nude mice were randomized into two groups. A2780 cells pretreated with SNHG10 overexpression or EV were subcutaneously injected into the mice. The tumors of the SNHG10 overexpression group were significantly smaller than those of the EV group (Fig. 8A). After 5 weeks, the tumors were harvested and weighed. The tumor weights of SNHG10 overexpression group were significantly reduced than the EV group (Fig. 8B). The above results indicated that SNHG10 overexpression inhibited the growth of the tumor in vivo. Then, we performed IHC to verify the correlation between SNHG10 and BIN1 in vivo. Consistent with the IHC results of human EOC specimens, BIN1 staining was mainly located in the cell nucleus. SNHG10 overexpression significantly upregulated the expression of BIN1 in vivo (Fig. 8C). Moreover, SNHG10 overexpression remarkably upregulated E-cadherin and downregulated N-cadherin in vivo (Fig. 8C). In summary, SNHG10 suppressed EOC tumorigenesis and progression in vivo by upregulating BIN1.

## Discussion

Recent studies indicate that lncRNAs play pivotal roles in the tumorigenesis and progression of multiple cancers [23]. LncRNAs act either as tumor-promoters or tumor-suppressors due to their precise downstream targets in cancer cells [6]. Numerous lncRNAs have been proven to be downregulated in EOC, among which some lncRNAs exhibit the potential to be novel biomarkers in diagnosis and prognosis prediction [24]. For instance, low expression of GAS5, promoting cisplatin-resistance in EOC cells, is significantly correlated with poor prognosis in EOC patients [25]. However, only a portion of lncRNAs have clear function in EOC, limiting further study to establish therapies targeting key lncRNAs. Thus, it is essential to reveal the functions and clinical significance of novel lncRNAs involved in the EOC tumorigenesis and progression.

By analyzing two EOC-correlated GEO datasets and overlapping the results, we screened out five candidate lncRNAs that were downregulated in EOC, among which SNHG10 exhibited the lowest expression. The effect of SNHG10 on tumorigenesis and progression varies in different cancers. In hepatocellular carcinoma, SNHG10 upregulates the expression of c-Myb and promotes the proliferation, migration, invasion and EMT of cancer cells [26]. Additionally, SNHG10 upregulates frizzled class receptor 3 (FZD3) to maintain the activation of the Wnt/β-catenin signaling pathway in osteosarcoma [27]. Moreover, SNHG10 facilitates malignant behaviors and stemness of glioma cells by targeting the miR-532-3p/F-box and leucine rich repeat protein 19 (FBXL19) axis [28]. In contrast, SNHG10 is downregulated in NSCLC, and low expression of SNHG10 predicted a poor prognosis [29]. In addition, SNHG10 upregulates sirtuin 1 (SIRT1), a tumor suppressor, and decreases the proliferation of NSCLC [30]. To our knowledge, the expression and clinical significance of SNHG10 in EOC remain elusive. In this study, we found SNHG10 was expressed at low levels in EOC tissues, indicating that SNHG10 might be a potential tumor-suppressing lncRNA. Furthermore, Kaplan–Meier analysis and COX proportional hazard progression model demonstrated that low expression of SNHG10 was correlated with a poor postoperative prognosis in EOC patients. These results indicated that SNHG10 might be a novel biomarker to predict the prognosis of EOC patients. To reveal the biological functions of SNHG10 in EOC, we overexpressed SNHG10 and evaluated its effects on the malignant behaviors of EOC cells. As a result, SNHG10 overexpression significantly inhibited the proliferation, colony formation, migration and invasion in EOC cells. Meanwhile, we knocked down SNHG10 expression to further confirm its tumor-suppressing effect in EOC cells. However, knocking down SNHG10 expression had no significant effect on the proliferation, migration, and invasion of A2780 and SKOV3 cells. A high abundance is essential for a lncRNA to exert biological functions [31], thus a further reduction might have no significant effect on the malignant behaviors in EOC.

To study the possible functional mechanisms of SNHG10 in EOC, we isolated the nuclear and cytoplasmic fractions and we found SNHG10 predominantly located in the cytoplasm. Acting as a ceRNA to sponge miRNAs and thereby regulating the expression of target genes is one of the most frequently functional mechanisms of cytoplasmic lncRNAs [32]. Thus, we used the bioinformatics algorithm DIANA LncBase v.2 experimental module to predict the miRNAs that were potentially sponged by SNHG10. The expressions of the top 10 candidate miRNAs, ranked by predicted score, were detected after overexpressing SNHG10. Among these 10 miRNAs, 5 miRNAs were downregulated by SNHG10 overexpression in EOC cells, and miR-200a-3p exhibited the most significant downregulation. miR-200a-3p belongs to the miR-200 family, whose biological significance and functional mechanisms in EOC are still disputed. Some studies demonstrate that miR-200a-3p is a tumor-suppressor in EOC and a biomarker predicting better prognosis [17]. Hu et al. [33] found that high expression of miR-200a-3p was significantly correlated with a lower recurrence rate and longer OS in EOC. Park et al. [34] found that miR-200a-3p suppressed EMT by targeting zinc-finger E-box binding homeobox 1 (ZEB1) and ZEB2 in numerous cancer cell lines. In contrast, Choi et al. [16] found that miR-200a-3p was highly expressed in EOC and correlated with the poor prognosis. Teng et al. [35] verified that high expression of miR-200a-3p was reliable for diagnosing EOC patients. Additionally, they performed Kyoto Encyclopedia of Genes and Genomes (KEGG) analysis and found that miR-200a-3p was significantly correlated with EMT, although its specific downstream target was not clear. Furthermore, Shi et al. [36] found that miR-200a-3p significantly promoted the proliferation, colony formation and invasion of EOC cells. In the present study, we observed that the expression of miR-200a-3p was significantly higher in EOC cells than in IOSE80 cell. Furthermore, we downregulated miR-200a-3p with miRNA inhibitor and subsequently found that the malignant behaviors of EOC cells could be suppressed. Thus, our results provide evidence that miR-200a-3p acted as a tumor-promoting miRNA in EOC. Taken together, these findings indicated that SNHG10 might suppress the progression of EOC by sponging miR-200a-3p.

Then, we analyzed the bioinformatics algorithms PITA, miRanDa and miRWALK to determine the downstream target of miR-200a-3p. By overlapping the results, we found 635 genes exhibiting the potential to be regulated by miR-200a-3p, among which BIN1 caught our attention due to our previous studies. BIN1 is a well-studied tumor suppressor that expressed at low levels or even lost in numerous cancers, including NSCLC, ESCC, oral squamous cell carcinoma (OSCC), melanoma, and breast cancer [21, 37]. The classical role of BIN1 in cancers is suppressing c-Myc’s function and activating caspase-independent cell death [38]. Our previous studies confirmed that BIN1 was expressed at low levels in NSCLC, resulting in c-MYC and EGFR dependent upregulation of PD-L1 [19]. Similarly, we determined that BIN1 was lowly expressed in ESCC and positively correlated with the postoperative prognosis [18]. However, the function and upstream regulatory mechanisms of BIN1 in EOC remain elusive. In this study, we found that BIN1 was expressed at low levels in EOC tissues, and low expression of BIN1 was correlated with a poor prognosis. Furthermore, we confirmed a SNHG10/miR-200a-3p/BIN1 axis in EOC cells by performing miRNA rescue experiments. Kaplan–Meier analysis showed that EOC patients harboring high expression of SNHG10 and positive expression of BIN1 had the best prognosis, compared with other groups of patients. These results indicated that it might be more favorable to detect the expression of SNHG10 and BIN1, simultaneously, to more precisely predict the prognosis of EOC patients. BIN1 exhibited a suppressive effect on EMT a critical process during EOC tumorigenesis and progression [20, 39]. EMT in epithelial cancer cells results in loss of cell-cell adhesion, and facilitates the transformation to mesenchymal stem cells with more invasive and migratory properties [40]. Loss of E-cadherin and overexpression of N-cadherin are considered to be fundamental events of EMT [41]. In this study, we found that the SNHG10/miR-200a-3p/BIN1 axis upregulated E-cadherin and downregulated N-cadherin, Twist and SNAIL, indicating that SNHG10 suppressed EMT in EOC cells.

In conclusion, our study verified the crucial biological function and clinical significance of SNHG10 in EOC. Low expression of SNHG10 was positively correlated with a poor prognosis in EOC patients. SNHG10 acted as a tumor-suppressor to inhibit tumorigenesis and EMT of EOC by regulating miR-200a-3p/BIN1. These findings lay the foundation for further clinical study on SNHG10/miR-200a-3p/BIN1 axis and provide novel insight into understanding of EOC tumorigenesis and progression.

## Materials and methods

### Patients and specimens

Eighty-five pairs of EOC specimens and matched fallopian tube specimens were obtained from patients who underwent cytoreductive therapy at the Fourth Hospital of Hebei Medical University (Shijiazhuang, China) between May 2015 and May 2016. All the tumor specimens were confirmed to be EOC by three independent pathologists. No patients received no antitumor therapy prior to surgery. The median patient age at the time of surgery was 59 years (range: 26–74 years). These patients were followed up every two months until death or 5 years after operation. All tissues were quickly placed in liquid nitrogen and preserved at −80 °C. This research was approved by the ethics committee of the Fourth Hospital of Hebei Medical University. All patients signed informed consent before surgery.

### Cell culture

The human ovarian cell lines A2780, SKOV3, OVCAR3, and OV90 and the normal human ovarian epithelial cell line IOSE80 were purchased from Procell Life Science & Technology Co., Ltd. (Wuhan, China). All cell lines were cultured in DMEM medium (Servicebio, Wuhan, China) containing 10% FBS (Solarbio, Beijing, China) and stored in a humidified incubator at 37 °C with 5% CO_2_. All the cell lines used in this study were re-authenticated by short tandem repeat (STR) profiling and tested for mycoplasma contamination in June 2021.

### Quantitative real-time PCR (qRT-PCR)

Total RNA from tissues and cells was extracted with TRIzol reagent (Invitrogen, Carlsbad, CA, USA), and cDNAs were synthesized according to the manufacturer’s instructions by using TaqMan MultiScribe Reverse Transcriptase (Applied Biosystems, Foster City, CA, USA). qRT-PCR analysis was performed using an ABI Prism 7900-HT Sequence Detection System (96-well, Applied Biosystems). Primers were designed and synthesized by RiboBio (Guangzhou, China). The primers used in qRT-PCR are listed in Supplementary Tables S1 & S2. The relative expressions of lncRNAs and mRNAs were normalized to GAPDH, and miRNAs were normalized to U6. The experiments were performed in triplicate.

### Cytoplasmic and nuclear fraction isolation

The cytosolic and nuclear protein fractions of EOC cells were collected by using the PARIS Kit (Thermo Fisher Scientific, Waltham, MA, USA). This experiment was performed according to the manufacturer’s instructions. GAPDH and U6 were used as internal controls for the cytoplasm and nucleus, respectively.

### Cell counting kit-8 (CCK-8) assay

For CCK-8 assays, cells were seeded into 96-well plates at a density of 1 × 10^3^ cells/well, and 10 μl of CCK-8 (Solarbio) reagent was added to each well. The cells were subsequently incubated at 37 °C for 2 h, and the absorbance was measured at 450 nm. Proliferation rates were determined at 24, 48, 72, and 96 h after transfection. The experiments were performed in triplicate.

### Colony formation assay

To detect the colony formation ability, 1 × 10^3^ EOC cells were inoculated into 6-well plates and cultured at 37 °C for 10–14 days. The colonies were then fixed with paraformaldehyde and stained with crystal violet. The number of colonies for each group was counted. The experiments were performed in triplicate.

### Wound-healing experiment and transwell assay

For the wound-healing experiment, EOC cells were seeded in 6-well plates for 24 h, and a line was scraped down the cell monolayer to introduce an artificial wound that was photographed at 0 h and 48 h. For transwell assays, transwell chambers (Corning, Corning, New York, USA) with a membrane pore size of 8 μm were coated with Matrigel (Solarbio). Subsequently, 4 × 10^4^ EOC cells were seeded in the upper chambers, and 600 μl medium supplemented with 10% FBS was placed in the lower chambers. After incubation for 24 h, the cells were fixed, stained, and counted using an inverted microscope. The experiments were performed in triplicate.

### Plasmid construction and cell transfection

To overexpress SNHG10 in EOC cells, a eukaryotic expression plasmid of human SNHG10 (full-length, NCBI Reference Sequence: NR_003138.3) was constructed using a pcDNA3.1 vector (GenePharma, Shanghai, China). The EV was used as negative control. In addition, siRNAs were used to knockdown SNHG10 in EOC cells. Sequences of siRNAs were as follows: si-SNHG10#1: sense: 5’- GCUUUGCAGUCGAGAUAUUGU-3’; si-SNHG10#2: 5’- GCAGUCGAGAUAUUGUUUAGC-3’. The cells were cultured in six-well plates for 48 h after transfection. qRT-PCR analysis was performed to confirm the transfection efficiency. miRNA mimics, miRNA mimic NC, miRNA inhibitors, and miRNA inhibitor NC were purchased from Thermo Fisher Scientific. To inhibit or overexpress indicated miRNA, the cells were transfected with matched inhibitor or mimic at a final concentration of 25 nmol/l. The cells were plated in 6-well plates for 24 h prior to transfection. The transfections were performed with Lipofectamine 2000 (Invitrogen, Carlsbad, CA, USA) according to the manufacturer’s instructions.

### Dual-luciferase reporter assay

The wild-type or mutant SNHG10 or BIN1-3’-UTR containing the predicted binding sites of miR-200a-3p was subcloned into an HBLumi Dual-luciferase reporter assay kit (Hanbio, Shanghai, China). The luciferase reporter plasmids were co-transfected into EOC cells with miR-200a-3p mimic or the negative control. The relative luciferase activity was measured according to the manufacturer’s instructions. All transfection assays were carried out in triplicate.

### RNA immunoprecipitation (RIP)

RIP assays were performed using the BersinBio^TM^ RIP Kit (BersinBio, Guangzhou, China) according to the manufacturer’s instructions. A2780 and SKOV3 cells were collected, and RIP lysate to obtain cell lysates. Anti-Ago2 antibody (ab186733, Abcam) or IgG (ab172730, Abcam) complex was prepared for immunoprecipitation. Then, the cell supernatants were incubated with magnetic bead-antibody complexes for 2 h at 4 °C. Next, RNA was purified, and the obtained RNA was used to detect the enrichment of SNHG10 and miR-200a-3p by conducting qRT-PCR.

### Western blotting

Cell lysates were separated by SDS-polyacrylamide gel (4%–10%) electrophoresis, and then transferred to polyvinylidene fluoride (PVDF) membranes (Millipore, Billerica, Massachusetts, USA). The membranes were then blocked with 5% skimmed milk and incubated overnight at 4 °C with the following primary detection antibodies: anti-BIN1 (14647-1-AP, Proteintech, Rosemont, USA), anti-E-cadherin (60335-1-Ig, Proteintech), anti-N-cadherin (66219-1-Ig, Proteintech), and anti-GAPDH (ab8245, Abcam). The species-matched secondary antibodies were then incubated for 2 h at room temperature and the proteins were detected using BeyoECL Plus (Beyotime, Shanghai, China). Each experiment contained triplicate wells of each sample, and all experiments were repeated at least three times.

### Fluorescence in situ hybridization (FISH)

For FISH assays, cells were first grown for 24 h in 24-well plates with glass cover slips. FISH probes were designed and synthesized by Servicebio. Biotin-labeled probes specific to SNHG10 and Dig-labeled locked nucleic acid miR-200a-3p probes were used in hybridization. Probe sequence: SNHG10: 5’-ACCGGGGTTCCAGCGCTCGGGCCGTAGCCT-3’, miR-200a-3p: 5’- ACATCGTTACCAGACAGTGTTA-3’. FAM and Cy3 were used to detect biotin-labeled and Dig-labeled probes, respectively. DAPI was used to counterstain the nuclei. Images were observed with a fluorescence microscope (Nikon, Tokyo, Japan).

### Immunohistochemistry (IHC)

IHC analysis was performed as we described in our previous study [42]. A rabbit polyclonal antibody against BIN1 (14647-1-AP, Proteintech) was used for detection. DAB was used for re-staining. BIN1 staining was evaluated by using IHC Profiler of ImageJ. Staining scores of 0 and 1+ were regarded as negative expression while 2+ and 3+ were regarded as positive expression.

### Xenograft tumor mouse model

A total of 20 four-week-old female BALB/c nude mice were purchased from the Animal Center of Huafukang (Beijing, China). All in vivo experiments were approved by the Committee on the Ethics of Animal Experiments of Hebei Medical University. Mice were randomly divided into two groups for injecting EOC cells. To establish xenograft tumor mouse model, A2780 cells (5 × 10^6^) transfected with SNHG10 or EV were injected into the axilla of the mice, and the tumor size (length; L, and width, W) was measured per week. Tumor volume was calculated by using the formula: 1/2 × *L* × *W*^2^. Five weeks later, the mice were sacrificed, and the tumors were harvested and weighed. No blinding was done in the animal experiment.

### Statistical analysis

The investigators were not blinded to the group allocation. The statistical analyses were performed using SPSS 26.0 software. Statistical graphs were drawn by using GraphPad Prism 8.0. All measurement data were presented as mean ± SD. The measurement data fitting the normal distribution were analyzed with student *t*-test, otherwise analyzed with Wilcoxon rank sum test. The correlations between SNHG10 expression and various clinicopathological were calculated by Spearman correlation analysis. Kaplan–Meier analysis and COX proportional hazards models were used to analyze the effects of variables on survival. Spearman correlation analysis was used to calculate the correlation between SNHG10 and miR-200a-3p/BIN1 expression. All tests were two-tailed, and *P*-value of <0.05 was considered to indicate statistical significance.

## Supplementary information

Supplementary Figure 1

Supplementary Figure 2

Supplementary Figure 3

Supplementary figure legends

Primer sequences of lncRNA and mRNAs for qRT-PCR

Primer sequences of miRNAs and U6 for qRT-PCR

Upregulated lncRNAs in GSE135886 and GSE119054 dataset

Predicted miRNA for binding with SNHG10 in LncBase v.2 experimental module

Downstream genes of miR-200a-3p, predicted by algorithms

## Data Availability

All data in this study are available upon reasonable request.
