# Supplementary figures and images for: Long non-coding RNA SNHG10 upregulates BIN1 to suppress the tumorigenesis and epithelial–mesenchymal transition of epithelial ovarian cancer via sponging miR-200a-3p
Source: Cell Death Discov. 2022 Feb 11;8:60. doi: 10.1038/s41420-022-00825-9 (PMC8837780; doi:10.1038/s41420-022-00825-9)

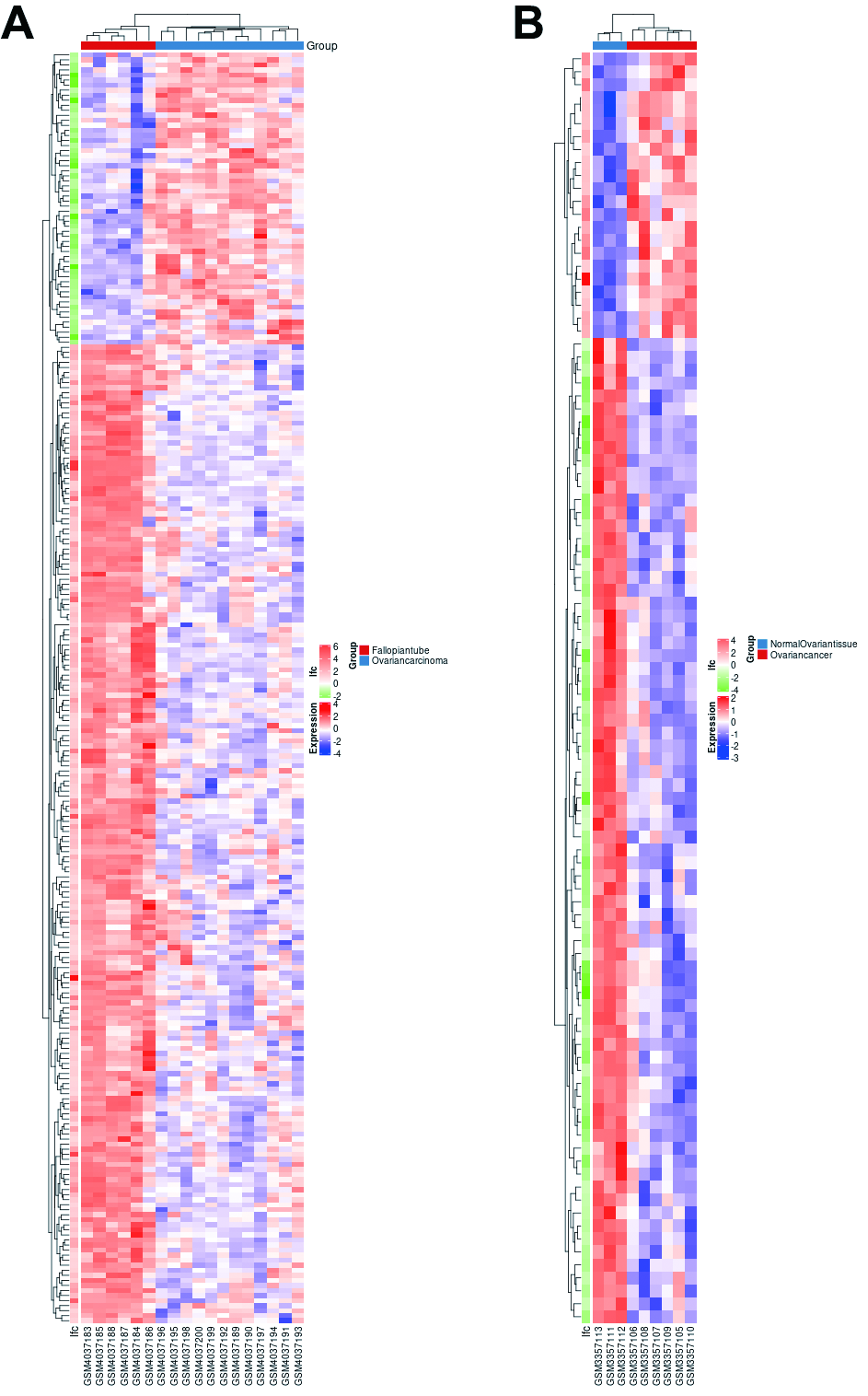

Supplement: Supplementary file 1 — Supplementary Figure 1 [file 41420_2022_825_MOESM1_ESM.tif]

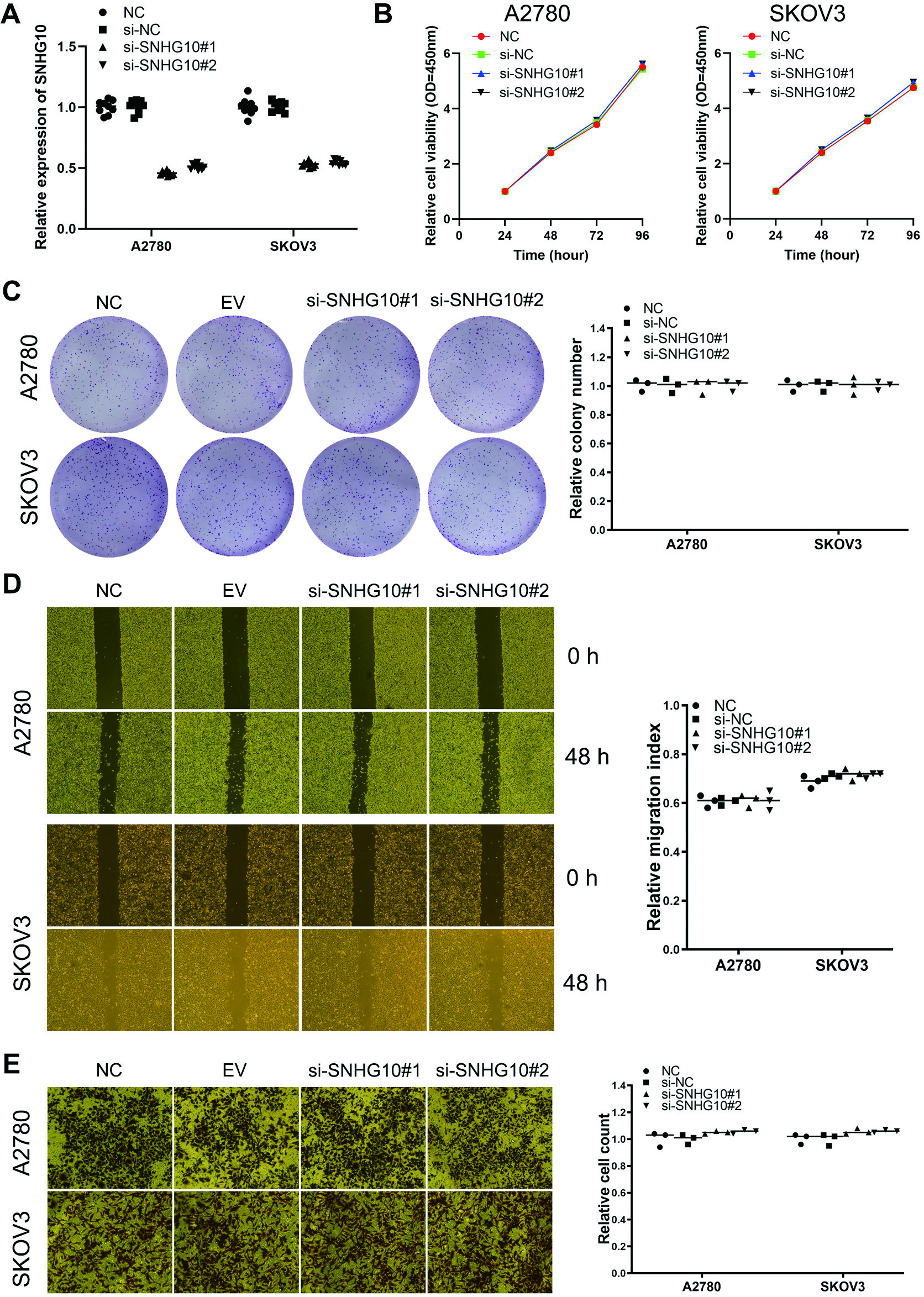

Supplement: Supplementary file 2 — Supplementary Figure 2 [file 41420_2022_825_MOESM2_ESM.tif]

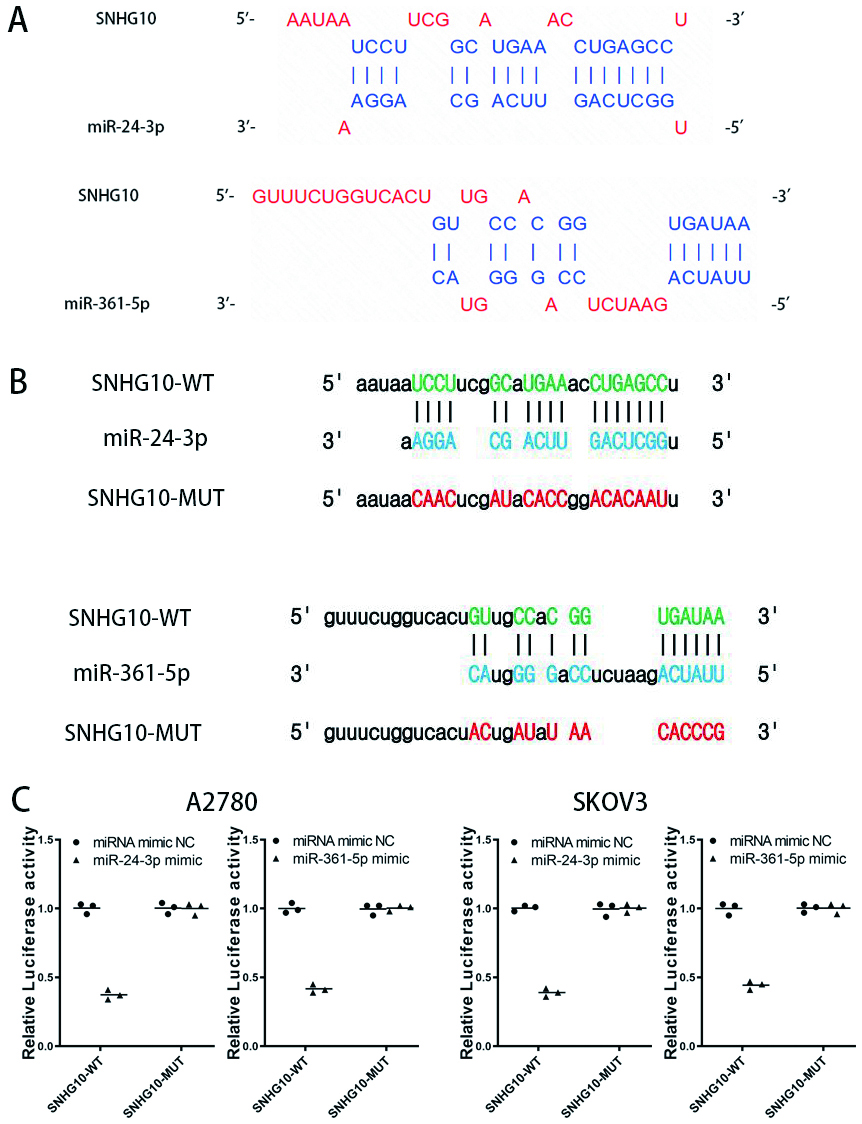

Supplement: Supplementary file 3 — Supplementary Figure 3 [file 41420_2022_825_MOESM3_ESM.tif]
