# Supplementary material for: Long non-coding RNA SNHG10 upregulates BIN1 to suppress the tumorigenesis and epithelial–mesenchymal transition of epithelial ovarian cancer via sponging miR-200a-3p
Source: Cell Death Discov. 2022 Feb 11;8:60. doi: 10.1038/s41420-022-00825-9 (PMC8837780; doi:10.1038/s41420-022-00825-9)
Supplement: Supplementary file 4 — Supplementary figure legends [file 41420_2022_825_MOESM4_ESM.docx]

**Supplementary Fig. 1 Heatmap of differential lncRNAs in GSE135886 and GSE119054 dataset**

**A** Heatmap of differential lncRNAs in GSE135886 dataset. **B** Heatmap of differential lncRNAs in GSE119054 dataset.

**Supplementary Fig. 2** **Effects of SNHG10 knockdown on the proliferation, migration and invasion of EOC cells in vitro.**

**A** Knockdown efficiency of siRNAs on the expression of SNHG10 in EOC cells. **B** Effect of SNHG10 knockdown on proliferation ability of EOC cells, assayed by CCK-8 method. Plots indicate mean. **C** Effect of SNHG10 knockdown on colony formation ability of EOC cells. **D** Effect of SNHG10 knockdown on migration ability of EOC cells, assayed by wound-healing experiment. **E** Effect of SNHG10 knockdown on invasion ability of EOC cells, assayed by transwell. The experiments were conducted in triplicate.

**Supplementary Fig. 3 Interaction between SNHG10 and miR-24-3p/miR-361-5p**

**A** The predicted binding between miR-24-3p and SNHG10, analyzed with LncBase v.2 experiment module. **B** The sequences of SNHG10-WT and SNHG10-MUT used in luciferase assay of miR-24-3p/miR-361-5p and SNHG10. **C** The miR-24-3p mimic and miR-361-5p mimic, respectively, decreased the luciferase activity of EOC cells transfected with SNHG10-WT but not in the cells transfected with SNHG10-MUT.
