# Supplementary material for: Long non-coding RNA SNHG10 upregulates BIN1 to suppress the tumorigenesis and epithelial–mesenchymal transition of epithelial ovarian cancer via sponging miR-200a-3p
Source: Cell Death Discov. 2022 Feb 11;8:60. doi: 10.1038/s41420-022-00825-9 (PMC8837780; doi:10.1038/s41420-022-00825-9)
Supplement: Supplementary file 5 — Primer sequences of lncRNA and mRNAs for qRT-PCR [file 41420_2022_825_MOESM5_ESM.docx]

Supplementary Table S1 Primer sequences of lncRNA and mRNAs for qRT-PCR

| Gene | Refseq | Sequence (5'-3') |
| --- | --- | --- |
| SNHG10 | NR_003138.3 | Forward: AGAGAAGCTTTGCAGTCGAGA  Reverse: TTCGGGTAATGTGCCGAGTT |
| PGM5P3-AS1 | NR_121189.1 | Forward: TGGTATTCACGCAGTTCCCA  Reverse: CGGACAGGCTGAAAGTACCAT |
| LINC01354 | NR_038426.1 | Forward: AGAGACCCCTCCAACGTTCT  Reverse: CAGAGACACACAGGGGACTG |
| LINC02593 | NR_122045.1 | Forward: TCCACGTCAGCTCACTCTCA  Reverse: TGTTACGCACGTTCTGGTCT |
| HOXA-AS2 | NR_122069.1 | Forward: CACGCTTTTCCCGTAGGAAG  Reverse: AGGTAAGCGCTGCTCCAAAA |
| GAPDH | NG_007073.2 | Forward: CCTGAGGGTTCTTTGTGCTGA  Reverse: AAAGGCTCAACCTTCCCCAT |
