# Supplementary material for: Long non-coding RNA SNHG10 upregulates BIN1 to suppress the tumorigenesis and epithelial–mesenchymal transition of epithelial ovarian cancer via sponging miR-200a-3p
Source: Cell Death Discov. 2022 Feb 11;8:60. doi: 10.1038/s41420-022-00825-9 (PMC8837780; doi:10.1038/s41420-022-00825-9)
Supplement: Supplementary file 6 — Primer sequences of miRNAs and U6 for qRT-PCR [file 41420_2022_825_MOESM6_ESM.docx]

Supplementary Table S2 Primer sequences of miRNAs and U6 for qRT-PCR

| Gene | Refseq | Forward Sequence (5'-3') |
| --- | --- | --- |
| miR-544a | NR_030257.1 | GGACGGTAGCAAGCAAAGAGTGTGCTCCTGTTCCT |
| miR-24-3p | NR_029496.1 | GGACGGTAGCAAGCAAAGAGTGTGATTAGCATCAC |
| miR-425-5p | NR_029948.1 | GGACGGTAGCAAGCAAAGAGTGTGTCAACGGGAGT |
| miR-4690-5p | NR_039839.1 | GGGATTCTGGAAGATGATGATGACGAGCAGGCGAG |
| miR-361-5p | NR_029848.1 | GGGATTCTGGAAGATGATGATGACGGAGCTTATCA |
| miR-34a-5p | NR_029610.1 | GGGATTCTGGAAGATGATGATGACGGCCAGCTGTG |
| miR-449a | NR_029960.1 | GGGATTCTGGAAGATGATGATGACCTGTGTGTGAT |
| miR-149-5p | NR_029702.1 | GGGATTCTGGAAGATGATGATGACGCCGGCGCCCG |
| miR-224-5p | NR_029638.1 | GGGATTCTGGAAGATGATGATGACGGGCTTTCAAG |
| miR-200a-3p | NR_029834.1 | GGGATTCTGGAAGATGATGATGACCCGGGCCCCTG |
| U6 | NR_138085.1 | ATTGGAACGATACAGAGAAGATT |
