# Supplementary material for: Long non-coding RNA SNHG10 upregulates BIN1 to suppress the tumorigenesis and epithelial–mesenchymal transition of epithelial ovarian cancer via sponging miR-200a-3p
Source: Cell Death Discov. 2022 Feb 11;8:60. doi: 10.1038/s41420-022-00825-9 (PMC8837780; doi:10.1038/s41420-022-00825-9)
Supplement: Supplementary file 8 — Predicted miRNA for binding with SNHG10 in LncBase v.2 experimental module [file 41420_2022_825_MOESM8_ESM.docx]

Supplementary Table S4 Predicted miRNA for binding with SNHG10 in LncBase v.2 experimental module

| miRNA | Pr. Score |
| --- | --- |
| miR-544a | 0.912 |
| miR-24-3p | 0.721 |
| miR-425-5p | 0.598 |
| miR-4690-5p | 0.593 |
| miR-361-5p | 0.520 |
| miR-34a-5p | 0.462 |
| miR-449a | 0.461 |
| miR-149-5p | 0.455 |
| miR-224-5p | 0.446 |
| miR-200a-3p | 0.415 |
